# Comprehensive In Vitro Profiling Demonstrates Successful Immune Evasion of Bioengineered Immune‐Shielded Heart Valves

**DOI:** 10.1002/adhm.71737

**Published:** 2026-09-21

**Authors:** Abraham van Wijk, Leanne Van den Berg, Isa B. M. Velraeds, Valentina Osorio Zuluaga, Emma T. M. Peereboom, Martijn J. W. E. Rabelink, Arnaud Zaldumbide, Rob C. Hoeben, Marie‐Jose T. H. Goumans, Eric Spierings, Carlijn V. C. Bouten, Marijn C. Peters

**Affiliations:** ^1^ Department of Paediatric Cardiothoracic Surgery, Wilhelmina Children's Hospital, Regenerative Medicine Center Utrecht University Medical Center Utrecht Utrecht the Netherlands; ^2^ Center For Translational Immunology University Medical Center Utrecht Utrecht the Netherlands; ^3^ Department of Cell and Chemical Biology Leiden University Medical Center Leiden the Netherlands; ^4^ Department of Biomedical Engineering Eindhoven University of Technology Eindhoven the Netherlands; ^5^ Institute For Complex Molecular Systems Eindhoven University of Technology Eindhoven the Netherlands

## Abstract

Living donor valves for valve replacement in pediatric patients, come with the risk of immune rejection of the implanted tissue. To address this, and increase the options for living implants in pediatric patients, we previously developed a lentiviral immune‐shielding strategy that leverages viral immune evasion mechanisms to downregulate surface HLA expression (via US2) and suppress natural killer (NK) cell activation (via Serpin b9). In this study, we comprehensively evaluated the impact of delivery of immune‐shielding genes in donor valve cells and whole donor valves on immune responses in vitro using host peripheral blood mononuclear cells (PBMCs). Our results demonstrate that this approach prevents immune cell‐mediated death of donor heart valve cells and reduces PBMC clustering around the allogeneic valve cells. Transcriptomic analysis of immune‐shielded donor valves via bulk RNA sequencing revealed upregulation of genes associated with immune tolerance pathways commonly observed in viral infections, cancer, and placental development. These findings support the impact of immune‐shielding technology as a strategy to protect allograft heart valves from immune‐mediated degeneration, advancing the path toward durable, immunologically compatible implants for pediatric patients.

## Graphical abstract

Living donor heart valves offer superior durability for pediatric patients but trigger immune rejection. Lentiviral delivery of viral immune‐evasion genes US2 and Serpin b9 into donor valve cells and valve tissue suppresses HLA expression, reduces PBMC clustering and cytotoxic killing, and induces transcriptional immune‐tolerance programs, paving the way toward durable, immunologically compatible living heart valve implants.

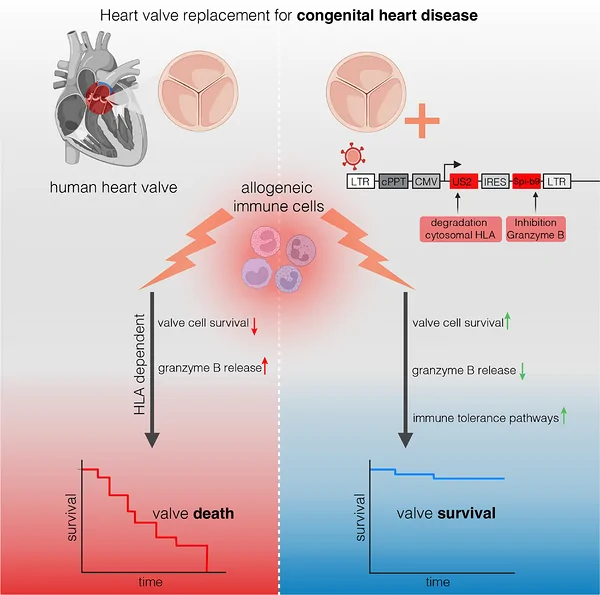

## 1 Introduction

Addressing irreparable heart valve dysfunction in young patients remains a significant challenge, as current heart valve grafts lack the ability to grow with the patient. Cryopreserved homografts from deceased donors are widely used but their inability to self‐repair or achieve growth in the developing heart necessitates multiple reoperations [1]. Encouraging outcomes from pulmonary autografts during the Ross procedure, where the patient's own pulmonary valve is used to replace a diseased aortic valve, as well as partial heart transplantation suggest that the key to durable implants in young patients lies in utilizing viable implants [2, 3, 4]. However, their implantation introduces the challenge of preventing immune activation. In partial heart transplantation, immunosuppressive medications are required in order to avoid prevent graft degeneration but this carries an increased risk of uncontrollable infections [5]. This limitation arises from the nonselective inhibition of immune responses during transplantation, where only graft‐specific immune activation leading to degeneration needs to be targeted [6]. Despite advances in immunosuppressive strategies, achieving immune tolerance of the graft remains rare. Approximately 80% of transplant recipients require long‐term maintenance on immunosuppressive drugs [7, 8]. These regimens combine agents from distinct pharmacological classes primarily targeting lymphocyte activation, including calcineurin inhibitors (e.g., tacrolimus and cyclosporine), which block T‐cell receptor‐mediated interleukin‐2 transcription, antiproliferative or antimetabolite agents (e.g., mycophenolate mofetil and azathioprine), which limit lymphocyte proliferation, rapamycin (mTOR) inhibitors (e.g., sirolimus and everolimus), and corticosteroids, often alongside induction therapies such as anti‐thymocyte globulin or IL‐2 receptor antibodies given around the time of transplantation [9, 10]. In pediatric heart transplantation specifically, tacrolimus‐based regimens combined with mycophenolate mofetil have become the golden standard of maintenance therapy, given evidence that CNI monotherapy alone is insufficient to optimize long‐term outcomes [11].

In cell transplants, ex vivo gene therapy to reduce immunogenicity has been explored and shown to achieve efficient immune tolerance in preclinical studies [12, 13, 14]. Specifically, downregulation of surface HLA class I and class II has enabled transplantation of pancreatic cells [15], mesenchymal stem cells [13], and induced pluripotent stem cells [12, 16, 17]. Similarly, viruses and cells at the fetal‐maternal interface achieve immune tolerance through the downregulation of surface HLA class and class II molecules. In particular, genes involved in inhibition of NK‐ and T‐cell responses, including HLA‐E [18, 19, 20, 21], HLA‐F [22, 23, 24], CD47 [12, 25, 26], CD55 [27, 28], CD46 [29], and CD200 [30, 31, 32, 33], as well as immunomodulatory genes such as HMOX1 [34, 35] and ISG15 [36, 37], have previously been implicated in immune tolerance across physiological and pathological settings. In addition, components of the immunoproteasome, including PSMB8 [38, 39] and PSMB9 [40, 41], regulate antigen processing for HLA class I presentation. Whether engineering valve cells with LV‐US2‐Spi9 induces such immune‐regulatory transcriptional programs remains unknown.

We have previously demonstrated the use of lentiviral delivery of herpesvirus immune evasion gene US2 and Serpin b9 to efficiently downregulate HLA class I and class II expression in human heart valve tissue, thereby reducing activation of HLA‐reactive CD8^+^ T cells [42].

Building on this work and leveraging our lentiviral immune evasion gene delivery system, we investigate the mechanisms of immune activation and immune cell‐mediated cytotoxicity in both untreated and treated donor heart valves. We analyse activation of blood‐borne immune cells after 7 days of coculture with donor heart valves to determine the full lymphocyte effector response, previously determined as the optimal time window and as the main driver of allograft rejection [43, 44, 45, 46, 47]. As a direct molecular readout of cytotoxic effector activity and a direct target of Serpin b9 [15, 48], Granzyme B was selected. Granzyme B is a serine protease released by cytotoxic T lymphocytes and NK cells upon contact with a donor cell. Once it is delivered intracellularly via perforin‐formed pores, it triggers caspase‐dependent apoptosis of the target cell. In the context of solid organ and heart transplantation specifically, Granzyme B expression in graft‐infiltrating lymphocytes has been shown to correlate with the severity of acute cellular rejection, supporting its relevance as a marker of alloimmune‐mediated graft injury [49]. Here, we conduct a comprehensive analysis of Granzyme B activity, IFN‐γ release, and valve cell death, in relation to HLA compatibility. This is complemented by transcriptomic profiling via RNA sequencing for an unbiased assessment of global gene expression changes following immune evasion gene delivery, compared to untreated heart valves. Our analysis includes isolated valve interstitial cells (VICs), valve endothelial cells (VECs), and whole donor valves.

## 2 Methods

### 2.1 Human Heart Valves

Aortic and pulmonary valves were obtained from patients undergoing surgical valve replacement at Wilhelmina Children's Hospital at Utrecht University Medical Center (Utrecht, the Netherlands). Tissue was obtained in accordance with the Declaration of Helsinki protocols and approved by the medical ethical assessment committee of Wilhelmina Children's Hospital at Utrecht University Medical Center (Utrecht, the Netherlands, TCBio number: 22–828). For pediatric valves, informed consent was obtained from parents or legal guardians. Additionally, male and female aortic and pulmonary valves were obtained from donor hearts processed by the Heart Valve Department of multi‐tissue bank ETB‐BISLIFE (Beverwijk, the Netherlands). Donor mediation was conducted by the Dutch Transplant Foundation (Leiden, the Netherlands), and permission for research was given for each donor. After removal from the aortic or pulmonary root, heart valve tissue was kept on ice in VIC medium (Dulbecco's Modified Eagle's Medium (DMEM) (Thermo Fisher #10313021) supplemented with 10% fetal bovine serum (FBS) (Sigma–Aldrich #F7524) and 1% penicillin/streptomycin (p/s)).

### 2.2 HLA Genotyping and PIRCHE Epitope Analysis

Unambiguous high‐resolution typing for HLA‐A, ‐B, ‐C, ‐DPA1, ‐DPB1, ‐DQA1, ‐DQB1, ‐DRB1, and ‐DRB3/4/5 was performed by applying a hybrid capture technology‐based next‐generation HLA sequencing protocol with the CareDx AlloSeq Tx17 kit following the instructions of the manufacturer (CareDx, Inc. – Brisbane, CA). Sequences were determined on the Illumina NovaSeq platform (www.illumina.com, Illumina, San Diego, USA). The PIRCHE‐T2 algorithm was employed to predict indirect allorecognition by estimating the number of epitopes derived from donor HLA molecules that could be presented by recipient HLA class II molecules. PIRCHE‐T2 were identified for each donor–recipient pair per experiment as previously described [50]. Only peptides with high binding affinities (predicted IC50 500 nM (PIRCHE‐I) or 1000 nM (PIRCHE‐T2) were accepted as relevant binders. PIRCHE‐I in the hematopoietic stem cell transplantation module was used to specifically identify class I presented peptides. Analyses were performed on the PIRCHE platform (PIRCHE AG, Berlin, Germany, available via www.pirche.com) [51, 52]. The algorithm uses donor HLA sequences to identify peptide epitopes and cross‐references these with recipient HLA class II molecules to estimate their binding likelihood. A higher PIRCHE‐T2 score indicates a greater predicted immunogenic potential of the graft. Subsequently, the PIRCHE‐B algorithm was used to predict B‐cell epitopes based on donor HLA sequences using the PIRCHE platform [53]. This algorithm evaluates the structural and antigenic properties of HLA molecules, estimating the potential for donor HLA to be recognized as foreign by recipient B‐cell receptors. The PIRCHE‐B analysis calculates the epitope load and provides a quantitative prediction of graft immunogenicity. Total PIRCHE‐B scores and PIRCHE‐T2 scores for HLA‐A, HLA‐B, and HLA‐C were analysed.

### 2.3 Valve Cell Culture

VICs and VECs were isolated as previously described [54]. Shortly, heart valve leaflets were incubated in 600U/mL collagenase II (Genaxxon Bioscience C4341.0100) supplemented VEC medium (EGMTM‐2 MV Microvascular Endothelial Cell Growth Medium‐2 BulletKit (Lonza #CC‐3202)) for 10 min at 37°C. VECs were gently scraped from the leaflet surface into a dish containing VEC medium using a sterile no.10 scalpel (Feather, #02.001.30.010) positioned at a 45° angle. Cells were washed twice with VEC medium by centrifugation at 400 g for 15 min and filtered through a 100 µm cell strainer. VECs were seeded on a fibronectin‐coated (Sigma–Aldrich #F0895) T25 flask. VICs were subsequently isolated by incubating the remaining leaflet in 600U/mL collagenase II in VIC medium for 20 h at 37°C. The cell suspension was then filtered through a 100 µm cell strainer and centrifuged twice at 400 g for 15 min before seeding in an uncoated T25 flask. Cells were detached at 80%–90% confluency using the Lonza detachment kit (Lonza #CC‐5034) and experiments were performed in passages 4 to 9. All the cell lines were regularly tested for mycoplasma contamination.

### 2.4 PBMC Isolation

PBMCs were isolated from donor blood of 4 healthy donors (Sanquin) after written informed consent according to the Declaration of Helsinki and in agreement with institutional guidelines. Density gradient centrifugation using Lymphoprep (Stem Cell # 18061) was used to isolate PBMCs and cells were maintained in VIC media. PBMCs were cocultured with valve cells at a 1:1 ratio and PBMC activation was measured using flow cytometry and ELISA after 7‐days coculture.

### 2.5 Lentiviral Transduction

DNA plasmid pLV‐CMV‐US2‐bc‐Spi9 was generated from two intermediate cloning vectors (pLV‐CMV‐US2‐bc‐GFP [55] and pLV‐CMV‐Spi9‐bc‐GFP [15]) as previously described [13, 15, 42].In short, pJET‐Spi9 vector has been generated by cloning a Spi9 cDNA PCR fragment into pJET2.1/blunt vector (Fermentas, St Leon‐Rot, Germany) using the following primers: Spi9 Fw 5′‐TACATAAGGTTACACTAT‐3′ and Spi9 Rv 5′‐AACCCTTGTGTTAAGTAA‐3′ on human placental cDNA (Agilent Technologies, Santa Clara, CA). Using a NotI/XbaI Spi9‐containing fragment, pJET‐Spi9 was cloned into a pLV‐CMV‐bc‐GFP vector. To generate the combined vector, the GFP fragment from pLV‐CMV‐US2‐bc‐GFP was removed after NsiI/MscI digestion and blunting reaction and replaced by the SmaI/SmaI Spi9‐encoding fragment of pLV‐CMV‐Spi9‐bc‐GFP. Following plasmid production, lentiviruses were produced in 293T cells with a third‐generation production system as previously described [56]. Viral yields were determined using antigen capture ELISA on HIV p24 levels (Zeptometrix #801002). An amount of 1 ng p24 was assumed to be the equivalent of 2 × 10^3^ transducing units (TU) and transductions were performed at a multiplicity of infection (MOI) of 2 TU/cell in medium supplemented with 8 ug/mL polybrene (R&D Systems #7711). Directly after transduction, plates were centrifuged at 600 g for 30 min at room temperature. Valve tissue was transduced at 3,2 ng/mm^3^ virus in the presence of 8 µg/mL polybrene for 24 h before medium was changed. Medium was changed every 2 days after transduction. Transduction efficiency was determined after 4 days by measuring HLA levels in flow cytometry and immunofluorescence staining.

### 2.6 Live–Dead Staining

Live–dead assay was performed using ethidium homodimer‐1/calcein AM staining with the Live/Dead Kit, for mammalian cells (Thermo Fisher Scientific #L3224) according to the manufacturer's instructions, and imaging was performed using the EVOS Floid microscope (Thermo Fisher Scientific #AMF5000). For live–dead assays, 3–5 fields of view per condition were imaged at fixed magnification using the EVOS Floid microscope. The number of viable, substrate‐attached valve cells (calcein‐AM‐positive) was quantified per field using ImageJ2, based on characteristic valve cell morphology (large, spread or elongated, adherent) to exclude cocultured PBMCs, which are small, round, and largely non‐adherent. As cells were seeded at equal density and imaged at fixed field size across all conditions, raw counts per field were used normalized to control counts of the same donor. For quantification of PBMC clustering, live and dead PBMCs in direct contact with a single valve cell were counted per field using the same morphological criteria in reverse (small, round cells co‐localizing with a valve cell).

### 2.7 Immunostaining

Cells were fixed 7 days after coculture with PBMCs using 4% paraformaldehyde (PFA) (Sigma–Aldrich #158127). Cells were permeabilized in 1% Triton X‐100 (Sigma–Aldrich #9036‐19‐5) in phosphate‐buffered saline (PBS) for 10 min followed by 1 h blocking in 1% bovine serum albumin (BSA) (Thermo Fisher #10735078001). Primary antibodies in 1%BSA/PBS were incubated overnight at 4°C followed by 3 × 10″ washing in PBS and secondary antibody incubations. After 3 × 10″ washing, cells were mounted using Fluoromount‐G mounting reagent (Thermo Fisher #00‐4958‐02). Valve tissue was embedded in Tissue Tek O.C.T. compound (Sakura #4583) for cryo‐sectioning at 10 µm thickness with a cryostat (Thermo Fisher, Epredia CryoStar NX50 Cryostat). Tissue was subsequently fixed in acetone (Thermo Fisher #177170025), permeabilized in 1% Triton X‐100, blocked in 1% BSA/PBS, and incubated with primary antibodies in 1% BSA/PBS overnight at 4°C. After three washing steps in PBS, secondary antibodies were added in 1 mg/mL Hoechst 33342 (Thermo Fisher #62249) containing 1% BSA/PBS for 1 h at room temperature followed by three washing steps in PBS and mounting using Fluoromount‐G mounting reagent.

Primary antibodies used were: Vimentin (rabbit, Cell signaling 57415, 1:200), CD45 (mouse, Protein Tech #60287‐1, 1:100), CD3 (rabbit, Protein Tech # 17617‐1, 1:100), CD8 (mouse, Santa Cruz Biotechnology #sc‐1177, 1:100). Alexa‐555 and Alexa‐488 antibodies were used as secondary antibodies (Thermo Fisher, 1:200). Confocal microscope (Leica SP8, LASx software) imaging was used for cell and sectioned leaflets. Image analysis was performed using ImageJ2 [57, 58].

### 2.8 Flow Cytometry

Cells were gently dissociated using the Multi‐Tissue Dissociation Kit (Miltenyi Biotec #130‐110‐204) and fixed using the Inside Stain Kit (Miltenyi Biotec #130‐090‐477). Antibodies used were: Granzyme B‐PE‐Dazzle (1:100, biolegend #372216), CD8‐605 (1:100, biolegend # 344742), and CD45‐APC (1:100, BD bioscience # 340910).

Flow cytometric analysis was performed using FACS LSRII and FACS Diva software (BD Bioscience). Data analysis was performed using FlowJo analysis software.

### 2.9 ELISA

Granzyme B was measured using Human Granzyme B ELISA Kit (Thermo Fisher #BMS2027‐2) according to manufacturer's instructions. Consecutively, light absorption was measured using a Multiskan FC Microplate Photometer (Thermo Scientific #51119000).

### 2.10 RNA Sequencing

After RNA extraction, RNA quality was determined using Qubit RNA HS Assay Kit (Thermo Fisher Scientific) and the Qubit fluorometer. The RNA library was prepared using the NEXTflex Rapid RNA‐seq Kit (Bio Scientific) and sequenced using CEL‐seq (Illumina) to determine RNA expression. DEGs analysis was performed using DESeq2 and the Galaxy EU server [59]. Pairwise comparisons between groups were conducted by applying the Wald test of the negative binomial distribution to the log2 gene counts, and significance was determined using an adjusted *p*‐value < 0.05. Based on the hypergeometric distribution, pathway enrichment analyses of DEGs were performed to screen the significantly enriched terms using R (v 3.2.0) using the databases of Gene Ontology (GO) and EnrichR [60]. Because of the sensitive nature of the human RNA sequencing data collected for this study, requests to access the raw sequencing dataset from qualified researchers trained in human subject confidentiality protocols may be sent to the corresponding author.

### 2.11 Statistical Analysis

Heart valves from 11 donors from ETB‐BISLIFE and five paediatric donors from Wilhelmina Children's Hospital were used. Commercially available VEC lines from three donors were used. The number depicted in the individual figures represents the number of independent experiments performed with separate cell batches. Statistical analysis was performed using Prism 8 (GraphPad Version 10.2.2 (341)) software. Normality was assessed using a Shapiro–Wilk test. To compare two normally distributed groups, a student's *t*‐test was performed. For three or more groups and the assessment of one parameter, one‐way ANOVA statistical test was used. Dunnett's multiple comparison test was used as post hoc analysis to determine significance at *p* < 0.05. Results are shown as mean ± standard error of the mean (s.e.m.).

## 3 Results

### 3.1 LV‐US2‐Spi9 Transduced VICs Show Improved Protection Against Immune‐Mediated Cell Death

To investigate the immune response to control heart valves and heart valves transduced with immune‐shielding genes US2 and Serpin b9 (Spi9), we first isolated VICs and VECs from fresh adult and pediatric human heart valves (Figure 1a). We established an in vitro system to study immune cell behavior around nonself heart valve cells by coculture of control and LV‐US2‐Spi9 transduced VICs (the primary heart valve cell type) with PBMCs from a different donor (Figure 1b). We established 7 days of coculture as the optimal time point to determine alloreactive cytotoxicity of PBMCs to our heart valve cells (Figure S1a,b). Our previous study demonstrated transduction with LV‐US2‐Spi9 decreases surface HLA‐ABC, HLA‐DR, HLA‐DP, and HLA‐DQ levels [42]. Here, we could confirm transduction with LV‐US2‐Spi9 decreased but not completely diminished surface HLA levels using a polyclonal antibody against HLA‐A, HLA‐B, and HLA‐C (Figure 2a,b). PBMCs in monoculture remained viable during 7 days in DMEM‐based cell culture medium (Figure 1c). Although PBMCs remained largely viable in monoculture, a low level of background PBMC death is expected without stimulation, consistent with cytokine‐withdrawal‐induced apoptosis of unstimulated lymphocytes [61, 62]. Therefore, to distinguish PBMC cell death from valve‐cell‐directed cytotoxicity in our quantification, we performed cell counting of attached cells based on their valvular cell morphology (large, elongated, and adherent). To determine whether coculture of VICs with nonself PBMCs induced VIC death, we performed a live–dead staining and determined the number of viable attached cells in six different VIC‐PBMC donor combinations. While visually, changes were hard to distinguish, quantification showed PBMCs induced cell death of nonself VICs in three donor combinations (1, 2, and 3) as quantified as a decrease in attached cells, while no significant PBMC‐induced cell death occurred in the other three donor combinations (4, 5, and 6) (Figure 1d,e). Transduction with LV‐US2‐Spi9 effectively reduced PBMC‐induced valve cell death in VIC‐PBMC donor combinations 1, 2, and 3 while maintaining cell survival in the combinations 4, 5, and 6, similar to control.

**FIGURE 1:**
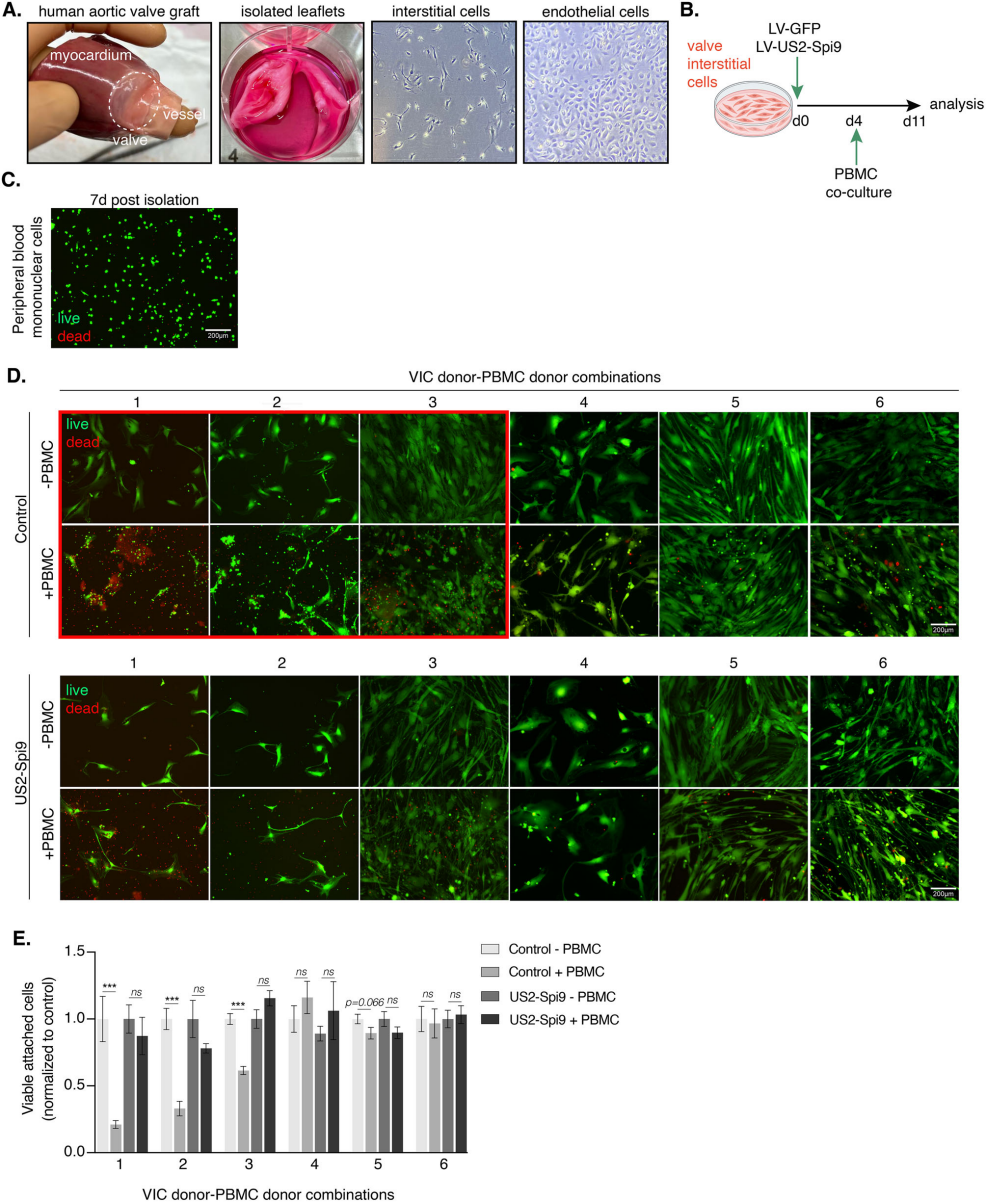
Immune cell‐induced valve cell death is dependent on donor–recipient combination. (A) Pipeline of heart valve cell isolation. (B) Schematic outline of experimental set‐up. (C) Peripheral blood mononuclear cells (PBMCs) remain viable in monoculture in DMEM‐based culture medium over 7 days. (D) Live (calcein AM)/dead (ethidium homodimer‐1) staining of six PBMC‐valve interstitial cell (VIC) cocultures after control or LV‐US2‐Spi9 transduction. (E) Quantification of (D). Data are shown as the mean ± SEM. ***p < 0.001 compared to control using unpaired, two‐tailed Student's *t*‐tests.

**FIGURE 2:**
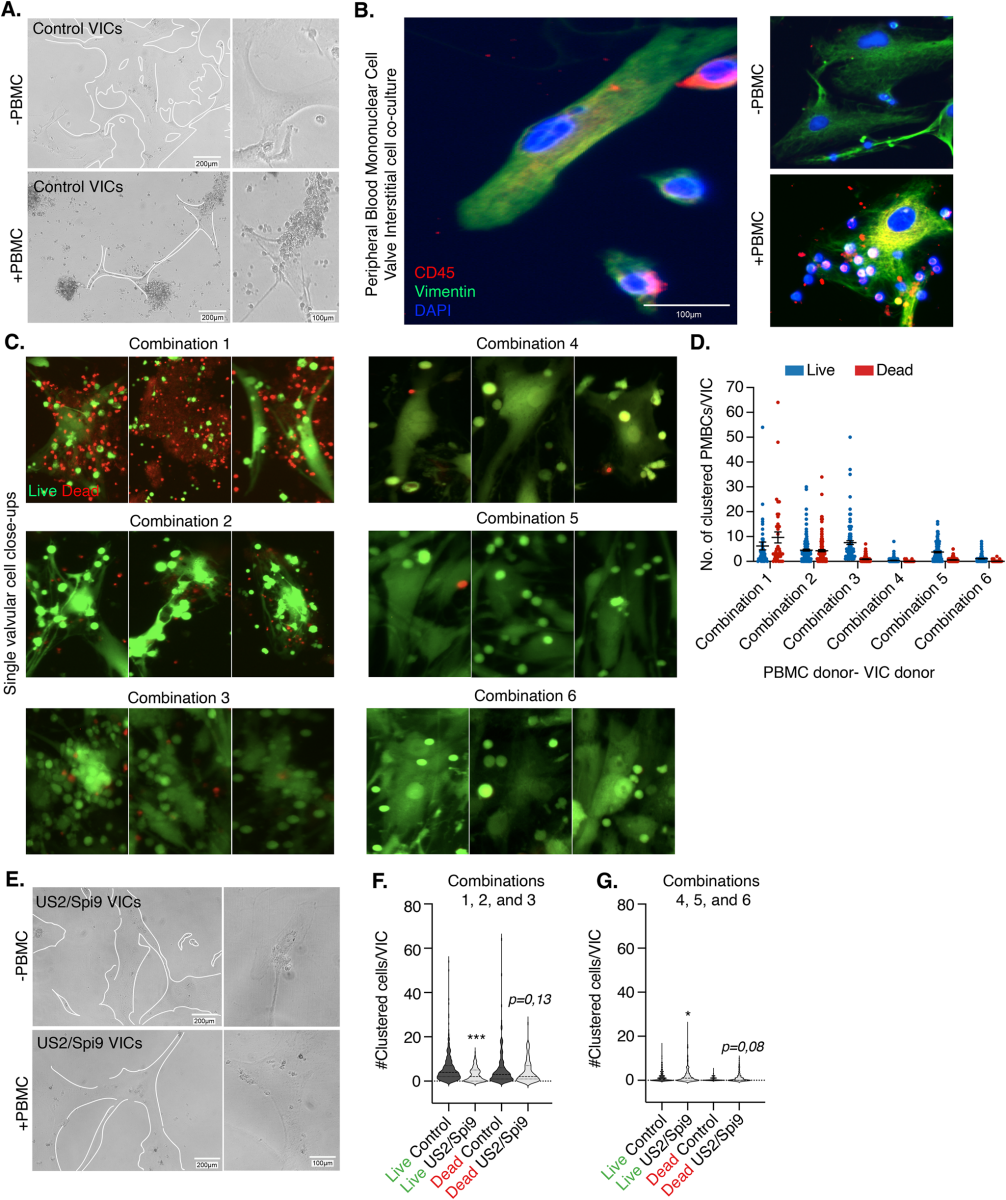
LV‐US2‐Spi9 prevents immune cell clustering around HLA‐mismatched VICs. (A) Representative bright‐field images of VICs in monoculture or VICs cocultured with PBMCs. (B) Immunofluorescence images of VIC in monoculture or coculture with PBMCs stained for CD45 (red), vimentin (Green), and dapi. (C) Close‐up of single cells of live (calcein AM)–dead (ethidium homodimer‐1) staining of six PBMC‐valve interstitial cell (VIC) cocultures. (D) Quantification of **C** showing number of clustered live (red) or dead (blue) PBMCs around a single valve cell. (E) Representative bright‐field images of LV‐US2‐Spi9‐transduced VICs in monoculture or cocultured with PBMCs. (F) Quantification of Live or dead clustered cells around single control or LV‐US2‐Spi9‐transduced VICs in donor–recipient combinations 1, 2, and 3. (G) Quantification of Live or dead clustered cells around single control or LV‐US2‐Spi9‐transduced VICs in donor–recipient combinations 4, 5, and 6. Data are shown as the mean ± SEM. * *p* < 0.05, ***p < 0.001 compared to control using unpaired, two‐tailed Student's *t*‐tests.

### 3.2 LV‐US2‐Spi9 Reduces PBMC‐Aggregation Around Nonself VICs

In the VIC‐PBMC cocultures, we observed the formation of cell clusters around the nonself VICs (Figure 2a). Immunostaining confirmed that these clusters were CD45^+^, indicating they consisted of PBMCs co‐localizing with the VICs (Figure 2b). Quantification of live and dead PBMCs around the valve cells revealed that in combinations 1, 2, and 3, up to 64 PBMCs clustered around a single valve cell. In contrast, in combinations 4, 5, and 6, the number of PBMCs co‐localizing with a valve cell remained below 16 (Figure 2c,d). Significantly reduced PBMC aggregation was found around US2/Spi9‐transduced valve cells in combinations 1, 2, and 3 compared to controls (Figure 2e,f). In combinations 4, 5, and 6, where no PBMC‐induced cell death was observed in controls, US2/Spi9 transduction did not reduce clustering and even slightly increased PBMC aggregation, without affecting valve cell viability (Figure 2g).

### 3.3 Immune Cytotoxicity and Rescue by LV‐US2‐Spi9 Correlate With HLA Mismatching

To investigate whether this observed difference between the immune cell response in combinations 1, 2, and 3 versus 4, 5, and 6 correlated with HLA compatibility between the immune cells and nonself VICs, we performed HLA genotyping and analyzed four categories: (1) shared HLA alleles in field 1 (e.g., HLA‐A*02), (2) shared HLA alleles in field 2 (e.g., HLA‐A*02:01), (3) shared HLA class I alleles in field 1, and (4) HLA class II alleles in field 1. The three combinations where coculture with PBMCs significantly reduced VIC viability (combinations 1, 2, and 3) exhibited a low to minor degree of HLA matching across all four categories (Figure 3a). Two of the three combinations without significant cell loss (combinations 4 and 6) demonstrated a moderate to high degree of HLA matching between PBMC‐donors and heart valve cells. Interestingly, in combination 5, where only a trend of PBMC‐induced cell death was observed, HLA typing showed a low to minor degree of HLA matching. A difference between combination 1, 2, 3, and 5 in HLA typing was found in an additional shared HLA‐A*01:01 allele.

**FIGURE 3:**
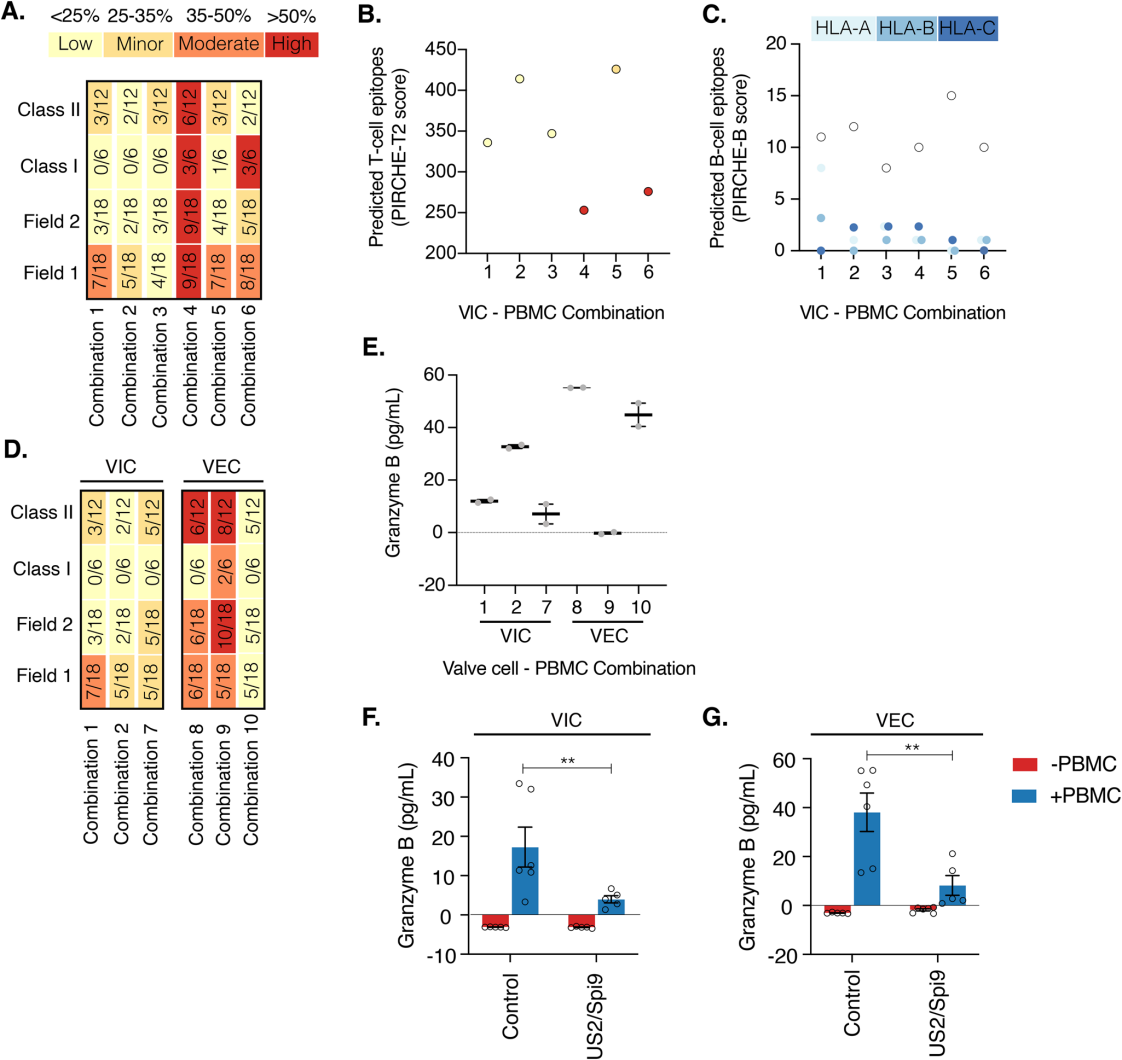
LV‐US2‐Spi9 protects against immune cell‐induced valve cell death and immune cell clustering and is linked to HLA compatibility. (A) HLA compatibility analysis of six PBMC‐VIC coculture combinations showing the number of common HLA alleles between VIC and PBMC donor divided by the total of alleles genotyped. Common alleles over field 1, field 2, HLA class I and HLA class II are depicted and categorized based on degree of overlap. Low: < 25% common HLA alleles; minor: 25%–35% common HLA alleles; moderate: 35%–50% common HLA alleles; high: > 50% common HLA alleles. (B) PIRCHE‐T2 scores of predicted T‐cell epitopes in six PBMC‐VIC combinations. (C) PIRCHE‐B scores of predicted B‐cell epitopes in six PBMC‐VIC combinations. Total PIRCHE‐B score and PIRCHE‐B scores of HLA‐A, HLA‐B, and HLA‐C are shown. (D) HLA compatibility analysis in three PBMC‐VIC and three PBMC‐VEC coculture combinations. (E) Comparison of secreted Granzyme B values between three PBMC‐VIC coculture combinations (1, 2, 7) and three PBMC‐VEC coculture combinations (8, 9, 10) (D). (F) Granzyme B levels in conditioned media of VIC monoculture of VIC‐PBMC cocultures after control or US2‐Spi9 transduction of the valve cells. (G) Granzyme B levels in conditioned media of VEC monoculture or VEC‐PBMC cocultures after control or US2‐Spi9 transduction of the valve cells. Data are shown as the mean ± SEM. ** *p* < 0.01 compared to control using unpaired, two‐tailed Student's *t*‐tests.

To further validate our observed connection between degree of HLA match and immune degeneration, we performed a deeper analysis of our HLA genotyping data through the Predicted Indirectly ReCognizable HLA‐Epitopes (PIRCHE) algorithm, commonly used in solid organ [63] and hematopoietic stem cell transplantation [50]. This algorithm shows computationally predicted HLA peptides from the valve donor that can be presented by HLA molecules of the immune cells to drive the immune destruction of foreign cells. The PIRCHE‐T2 algorithm, for epitopes presented on HLA class II strongly relates to anti‐HLA antibody formation [52] and antibody‐mediated [63] and T‐cell‐mediated rejection [50, 53, 64]. Analysis of PIRCHE‐T2 values in our valve–PBMC donor combinations showed high PIRCHE‐T2 values (> 276) across all combinations, indicating substantial CD4+ T‐cell capacity and indirectly recognizable HLA epitopes, potentially contributing to T‐cell activation (Figure 3b). In line with our HLA typing data and in vitro immune assay, the highest PIRCHE‐T2 values corresponded to the combinations 1,2, and 3 (that showed significant immune‐mediated cell death) and combination 5 (that showed a trend toward immune‐mediated cell death). As our in vitro assay does not measure antibody responses, we also analyzed predicted B‐cell epitopes using the PIRCHE‐B model. While PIRCHE‐IT2 values for T‐cell epitopes aligned with the observed immunogenicity in vitro, predicted B‐cell epitopes did not correlate with PBMC‐induced VIC death (Figure 3c). These findings suggest that our PBMC‐based in vitro immune assay specifically models T‐cell‐mediated responses.

To evaluate the effects of valve cell type (VIC vs. VEC) on immune cell cytotoxicity, we assessed Granzyme B levels in coculture media after 7 days in control and LV‐US2‐Spi9‐transduced VICs and VECs. As expected, in the VIC‐PBMC cocultures, the highest Granzyme B secretion was observed in the donor combination with the lowest degree in HLA matching, while the other two combinations with low to minor HLA matching showed similar levels of induced Granzyme B release (Figure 3d,e). Surprisingly, in the VEC‐PBMC combinations, combination 8 elicited the highest Granzyme B secretion but showed a higher degree of HLA allele matching (field 1, 2, class II) (Figure 3e, Figure S3a,b). This elevated Granzyme B release in combination 8 may be attributable to the absence of any HLA class I allele matches, consistent with the low Granzyme B levels observed in combination 9 which has less HLA class I mismatches. Most importantly for the efficacy of our therapeutic strategy, transduction with LV‐US2‐Spi9 significantly decreased Granzyme B release in cocultures of PBMCs with VICs and VECs, despite the observed variation in baseline Granzyme B levels in the differently HLA‐matched donor combinations (Figure 3f,g). To obtain a more complete profile of HLA compatibility and assess HLA epitopes that could lead to CD8^+^‐T cell responses, we used PIRCHE‐T1 analysis. PIRCHE‐T1 analysis did not show a link with class I presented HLA epitopes and PBMC‐induced heart valve cell death as the highest PIRCHE‐T1 values were found in the best HLA‐matched combinations (Figure S3c). This suggests indirect recognition of HLA class I‐derived peptides via class II presentation could be leading in triggering valve cell death, consistent with earlier observations in clinical kidney transplantation studies [53, 64]. Alternatively, direct recognition by CD8^+^‐ T‐cells could also play a role. In conclusion, exposing valve cells to PBMCs induces cytotoxic activation of PBMCs and valve cell death in donor combinations with lower degree of HLA matching, highlighting the interplay between immune recognition and valve cell immunogenicity.

Transduction with LV‐US2‐Spi9 can alleviate this cytotoxic activation of PBMCs as seen by decreased valve cell death and decreased Granzyme B release.

### 3.4 RNA Sequencing of Transduced VECs and VICs Reveals Conserved Immune Evasion Gene Signatures Induced by LV‐US2‐Spi9

In order to fully investigate the effect of transduction on the valve cells, we analysed the VIC and VEC transcriptome. Interestingly, transduction of VECs led to a higher number of differentially expressed genes (DEGs) than transduction of VICs with a fourfold increased number of upregulated genes (Figure 4a,b). Principal component analysis (PCA) of log fold‐change values revealed clear separation between US2/Spi9‐treated samples and controls in both VIC and VEC datasets indicating distinct transcriptional profiles (Figure S4a, b). Analysis of major GO biological terms according to their Z score revealed differentially expressed pathways involving regulation of the immune response, regulation of the viral process, regulation of the intrinsic apoptotic pathway (VECs) and the endoplasmic reticulum (ER) unfolded protein response (VICs) (Figure 4c,d, Table S1). The regulation of the intrinsic apoptotic pathway GO term only showed DEGs E2F1 and TNFRSF1B downregulated after transduction with LV‐US2‐Spi9 related to decreased immune‐induced cellular apoptosis. Interestingly, both pathways involving negative regulation of the immune response as well as pathways involving positive regulation of the immune response were found upregulated in LV‐US2‐Spi9‐transduced cells. The DEGs were then grouped into the four major clusters and the major GO biological terms involved in these clusters were noted in the heatmap. The heatmap revealed several DEGs involving negative regulation of T‐ and NK‐cells that were upregulated in LV‐US2‐Spi9‐transduced cells including ISG15‐USP18 [65], BIRC3 [66], HMOX1 [67], HLA‐F [24, 67], CD47 [12], CD55 [68], DDRGK1 [69], CD46 [29], NR1D1 [70], TMED7 [71], LGALS3 [26], CEACAM1 [72], DUSP3 [73], LAPTM [74], GBP1 [75], NCK1 [76, 77], TNFSF4 [78], ZBTB1 [79], AKT1 [80], and CD200 [32, 33], genes that were previously found to mediate immune evasion in stem cells, viruses, cancers and the placenta (Figure 4e,f). These findings were in line with downregulation of genes involved in positive regulation of T‐ and NK‐cell mediated immunity including CD248 [81], ALCAM [82], APOBEC3B [83], CREBBP [84, 85], CIITA‐regulated gene PLXNA1 [86], MAP3K7 [87], HCG15 [88], TNFRSF1B [89], LRRC14 [90, 91], AURKB [92], and RAB34 [93]. Additionally, we also observed LV‐US2‐Spi9‐induced upregulation of genes involving negative regulation of the viral process potentially relating to CMV promoter silencing. Unexpectedly, in VECs, DEGs also showed LV‐US2‐Spi9 induced upregulation of genes involving positive regulation of T cells among which HLA‐A and HLA‐B, which are direct targets triggered to be degraded by US2, and B2M, the beta‐chain of major histocompatibility complex class I molecules. To investigate whether this upregulation of HLA mRNAs leads to an increased presence of HLA‐A, HLA‐B, and HLA‐C proteins in the cell or at the cell surface, we performed immunostaining without permeabilization to solely show cell surface HLA levels and with permeabilization to also show intracellular HLA levels. These stainings indicated that, in line with our previous findings, cell surface HLA‐A, ‐B, and/or ‐C remain strongly decreased in LV‐US2‐Spi9 transduced cells while intracellular HLA proteins remained similarly present (Figure 5a,b). To investigate whether HLA molecules co‐localized with the ER after transduction with LV‐US2‐Spi9 we stained for calnexin. While control cells showed the presence of HLA‐A, ‐B, and/or ‐C throughout the cells and point localizations at the cell borders, co‐staining with calnexin confirmed cells with HLA accumulation in the ER after transduction with LV‐US2‐Spi9 (Figure 5c,d). This suggests ER retention as a working mechanism of immune evasion via US2/Spi9 delivery.

**FIGURE 4:**
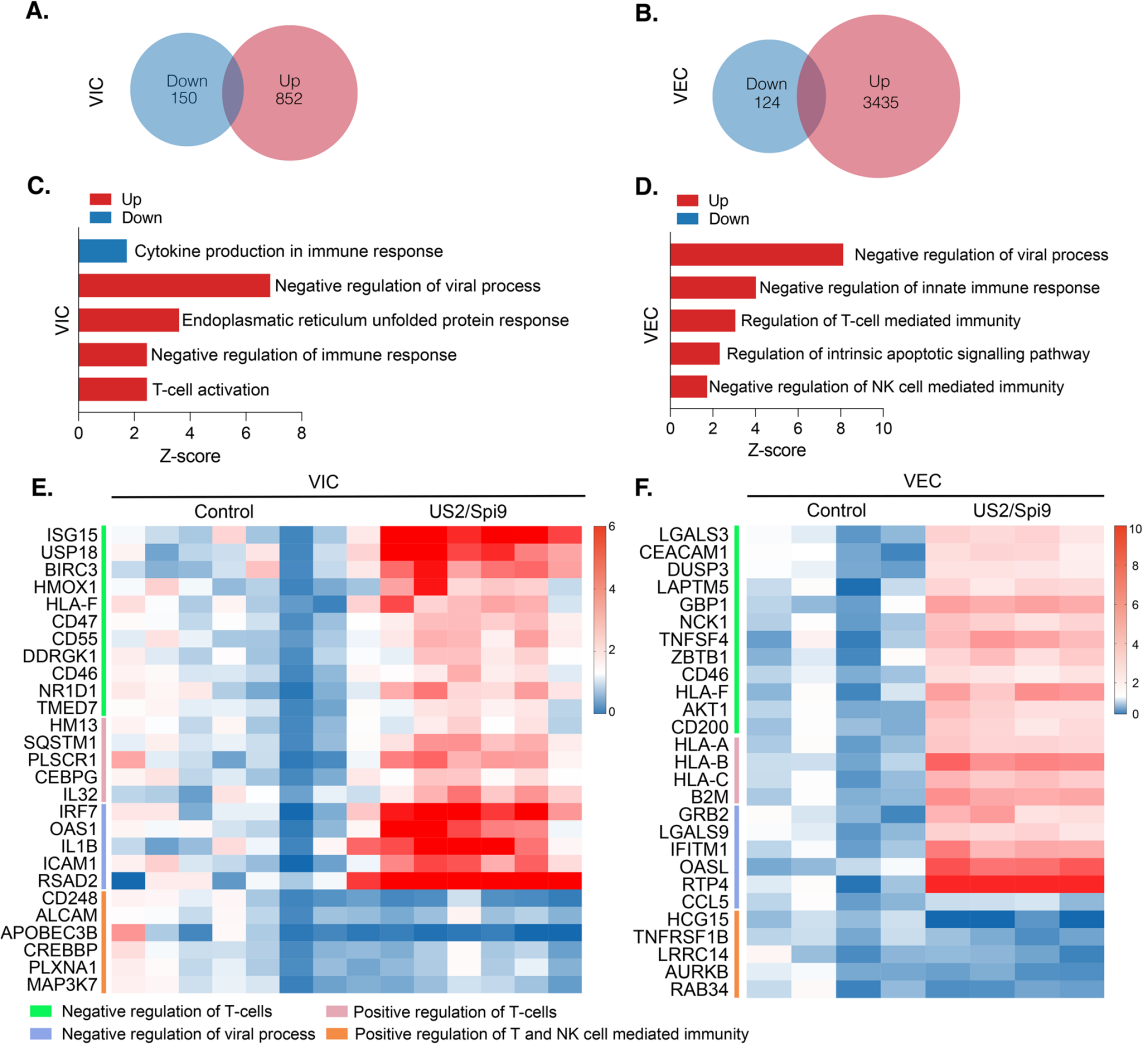
LV‐US2‐Spi9‐transduced VICs and VECs show conserved immune evasion gene signature. (A, B) Venn diagram showing the number of up‐ and down‐regulated differentially expressed genes (DEGs) after US2/Spi9 transduction compared to control cells in VICs (A) and VECs (B). (C, D) Gene Ontology (GO) analysis of DEGs between control and LV‐US2‐Spi9‐transduced cells, indicating the top upregulated (red) and downregulated (blue) biological processes enriched after transduction with LV‐US2‐Spi9 ranked by z‐score in VICs (C) and VECs (D). (E, F) Heatmap of normalized reads of top DEGs with main biological processes involved in control and LV‐US2‐Spi9‐transduced VICs (E) and VECs (F) analyzed by DESeq2. Fold change compared to control is shown.

**FIGURE 5:**
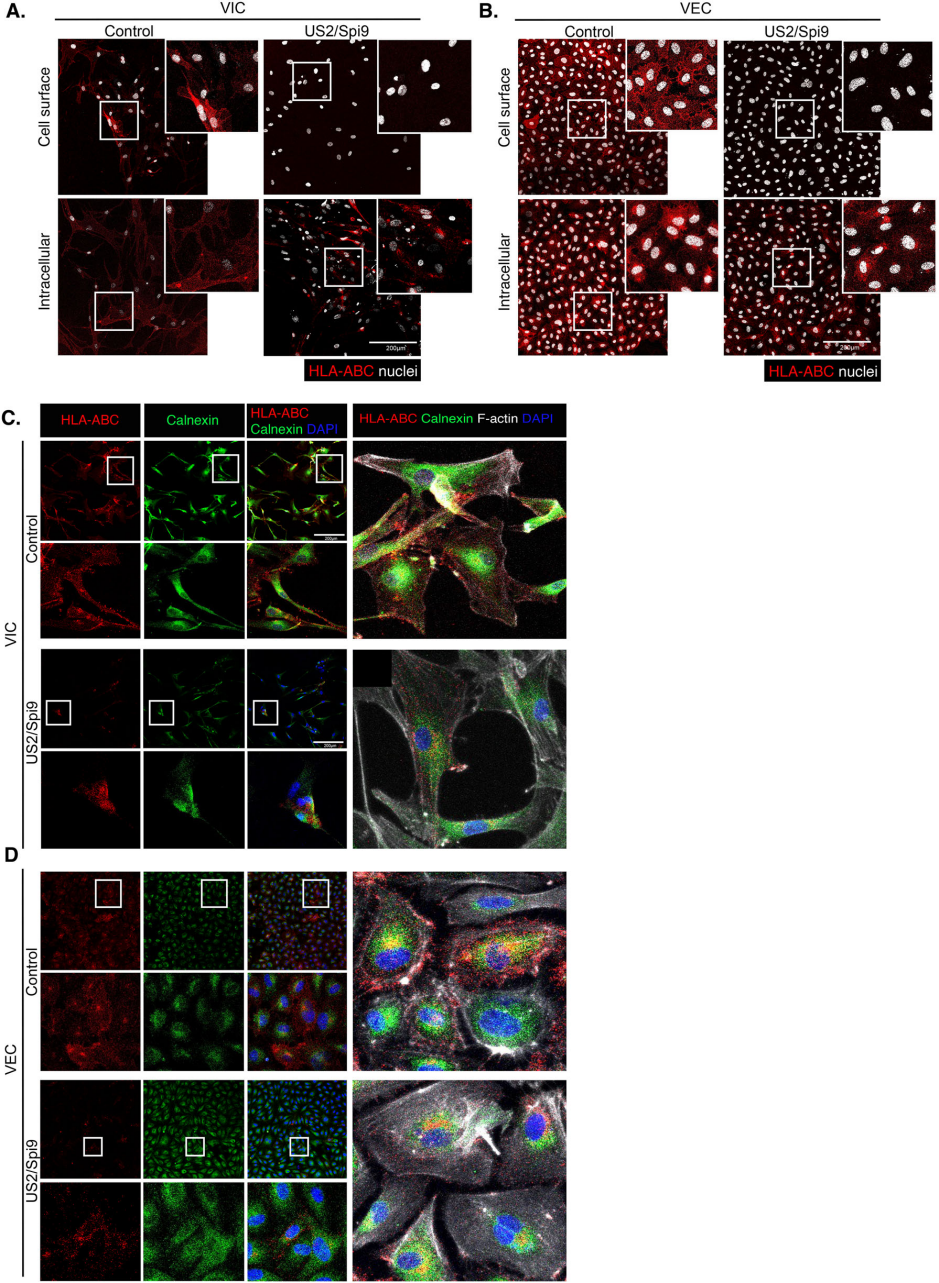
LV‐US2‐Spi9 strongly decreases surface HLA‐A, ‐B, ‐C levels while intracellular HLA appears accumulated in the ER. (A) Immunofluorescence images of VICs transduced with LV‐US2‐Spi9 or control without permeabilization solely showing cell surface‐bound HLA‐A, ‐B, and/or ‐C, and with permeabilization showing also intracellular HLA‐A, ‐B, and/or ‐C levels. (B) Immunofluorescence images of VECs transduced with LV‐US2‐Spi9 or control without permeabilization solely showing cell surface‐bound HLA‐A, ‐B, and/or ‐C, and with permeabilization showing also intracellular HLA‐A, ‐B, and/or ‐C levels. Square shows area of close‐up. (C, D) Immunofluorescence images of VICs (C) and VECs (D) transduced with LV‐US2‐Spi9 or control stained for calnexin (endoplasmic reticulum, green), HLA‐A, ‐B, and/or ‐C levels (red), and phalloidin (F‐actin, gray) showing HLA molecules after transduction colocalize with calnexin.

### 3.5 Decreased Immune Activation in Immune‐Shielded Donor Heart Valves

To evaluate the impact of LV‐US2‐Spi9 on immune activation in a tissue context, we cocultured intact heart valve leaflets with nonself PBMCs. Fresh pulmonary or aortic valve leaflets were isolated and directly transduced with LV‐US2‐Spi9 (Figure 6a). Four days post‐transduction, PBMCs were added to the cultures, and secretion of Granzyme B and IFN‐γ was measured in the supernatant as indicators of PBMC activation after 7 days. Consistent with our earlier VIC and VEC data showing donor‐dependent immune responses, tissue‐PBMC cocultures displayed variable levels of Granzyme B and IFNγ. Importantly, transduction with LV‐US2‐Spi9 led to a notable reduction in Granzyme B secretion, particularly in donor combinations that exhibited high baseline Granzyme B levels in controls (Figure 6b), indicating effective suppression of immune activation. A similar trend was observed for IFN‐γ (Figure 6c). Donor combinations 11, 12, and 13 exhibited low IFNγ release across all conditions. In contrast, combinations 14 and 15 showed high baseline IFNγ secretion in PBMC monocultures; however, coculture with heart valve tissue did not further increase IFNγ secretion in a cytotoxic response. In these combinations, where exposure to the heart valve did not induce IFNγ release, LV‐US2‐Spi9 transduction did not further reduce baseline IFN‐γ levels. In tissue donor‐PBMC combination 16, where exposure to PBMCs strongly increased IFNγ secretion in control tissues, transduction with LV‐US2‐Spi9 suppressed this response. These results demonstrate that LV‐US2‐Spi9 delivery to heart valve tissue can attenuate PBMC activation, thereby reducing immunogenicity. To determine whether PBMCs entered the valve tissue or became activated at the valve surface, we used cryosections of the tissue to determine their localisation within the tissue. Lymphocytes (CD3^+^ cells), including CD4^+^ and CD8^+^ T‐cells, and macrophages (CD68^+^ cells), were observed within the deeper valve layers (approximately 400 µm from the surface) (Figure 6d‐f). To determine the effect of transduction on the transcriptome of treated heart valve leaflets, we performed bulk RNA‐sequencing on the control and LV‐CMV‐US2‐Spi9 transduced human heart valve leaflets. Transduction of heart valve tissue resulted in fewer DEGs than in isolated VECs and VICs (Figure 7a). The number of downregulated and upregulated DEGs in LV‐CMV‐US2‐Spi9‐transduced heart valves was similar. In contrast to VECs and VICs, PCA revealed that sample clustering was primarily driven by the heart valve donor, indicating, donor‐specific transcriptional signatures dominate the variance in gene expression as opposed to transduction with LV‐CMV‐US2‐Spi9 in tissues (Figure S5). Analysis of major GO biological terms indicated differentially expressed pathways involving regulation of receptor internalization, neutrophil and monocyte‐mediated immunity, proteasome‐mediated catabolic process, regulation of the immune response, and protection from natural killer cell cytotoxicity (Figure 7b). Compared to the high enrichment strength as found in the VEC and VIC RNA sequencing data sets (Z‐scores > 2), GO term analysis of LV‐US2‐Spi9 transduced tissue showed moderate enrichment strength (Z‐scores 0–2) with only a small number of DEGs per GO term. The heatmap of the genes as clustered in these differentially regulated pathways indicated DEGs involving negative regulation of the immune response that were upregulated among which TRIM59 [94], PPFIA4 [95], HLA‐E [18, 20], HLA‐DOA [96], and KISS1 [97, 98] genes previously found to mediate immune evasion in cancers, viruses and/or the placental interface (Figure 7c). Consistent with the observed DEGs associated with upregulated HLA machinery in VECs and VICs, a modest upregulation of genes involved in immunoproteasome‐mediated catabolic processes was detected, including PSMB8 and PSMB9, which are key components in antigen processing for HLA class I presentation [38, 39]. DEGs involving the GO term regulation of receptor internalization were downregulated following LV‐US2‐Spi9 transduction among which GREM1 [99], APLNR [100], and UBXN10 [101] described to play a role in cancer immune evasion, and LRRK1 [102] previously found to play a role in regulating B‐cell responses. DEGs involved in positive regulation of immune process were also downregulated, including CHMP4A [103], SYTL2 [104], TNFSF18 [105, 106], ANXA3 [107], associated with lymphocyte activation, and FCAR [108] associated with B‐cell responses (IgA).

**FIGURE 6:**
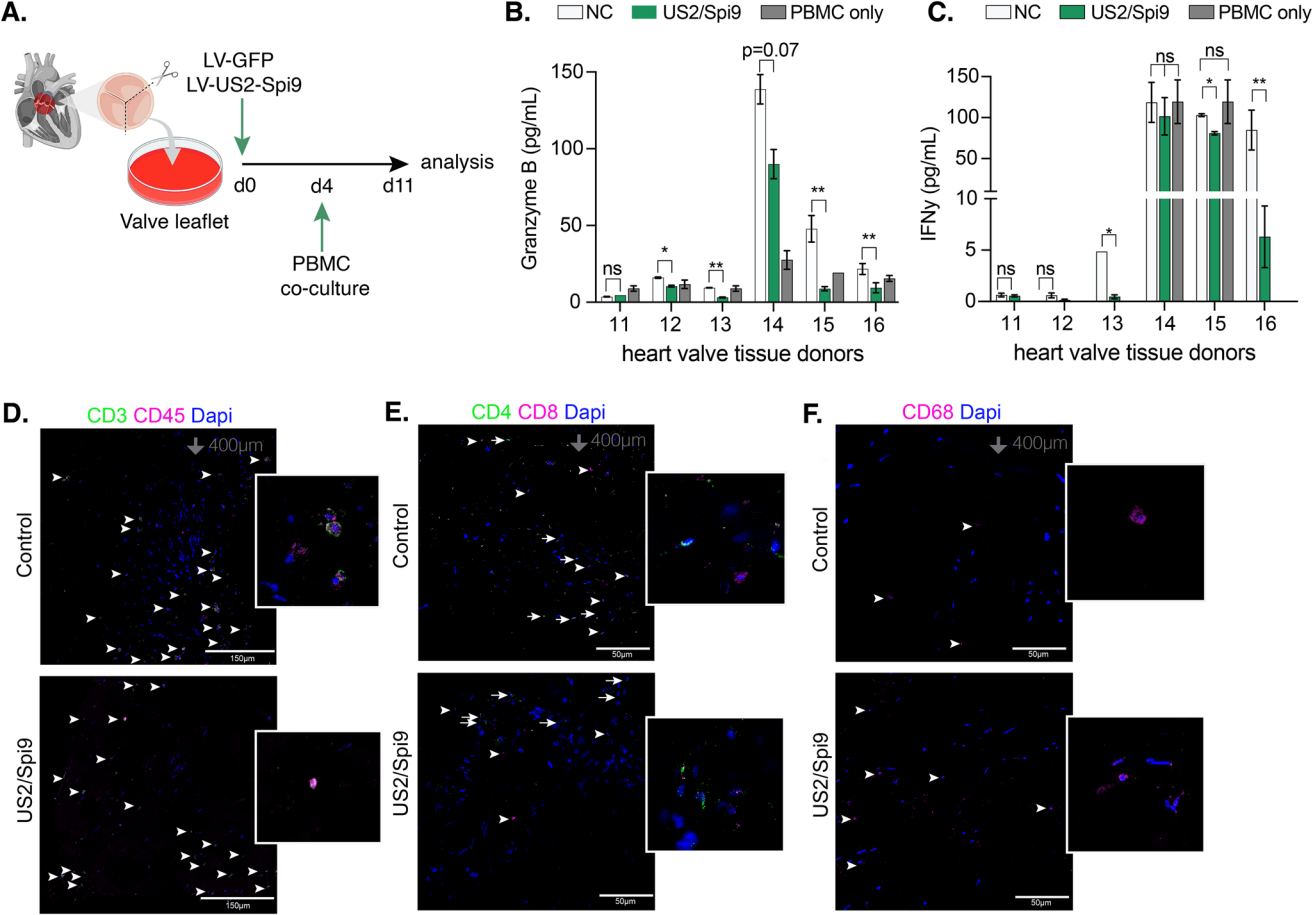
Decreased immune cell activation following US2/Spi9 transduction despite deep immune cell infiltration within the heart valve leaflets. (A) Schematic outline of experimental set‐up. (B, C) Secreted Granzyme B (B) and IFNy (C) levels in media of heart valve‐PBMC cocultures or PBMC monoculture in control or US2/Spi9 treated valve leaflets. Combinations indicate the variation in heart valve donor and PBMC donor used. (D–F) Immunofluorescence images of control and US2/Spi9 treated heart valves in coculture with PBMCs. CD45 stain indicated PBMCs; CD3 stain indicates T‐lymphocytes (D); CD8 stain indicatesCD8+ T‐lymphocytes; CD4 stain indicates CD4+ T‐lymphocytes; CD68 stain indicates macrophages (F).

**FIGURE 7:**
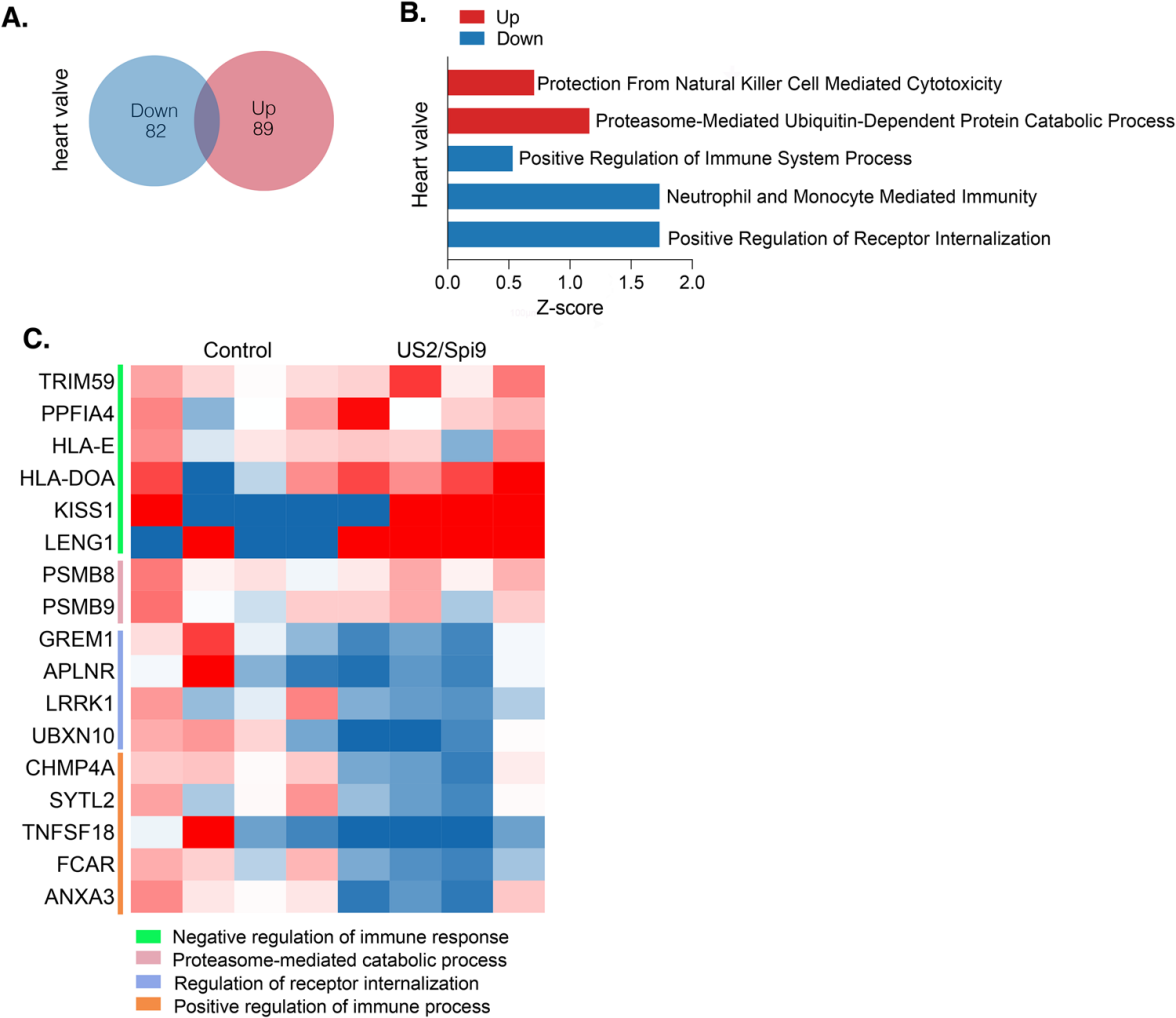
RNA sequencing identifies an immune shielding transcriptomic profile in US2/Spi9 transduced heart valves. (A) Venn diagram showing the number of up‐ and down‐regulated differentially expressed genes (DEGs) after US2/Spi9 transduction compared to control heart valves. (B) GO analysis of DEGs between control and LV‐US2‐Spi9‐transduced cells indicating the top upregulated (red) and downregulated (blue) biological processes enriched after transduction of heart valve leaflets with LV‐US2‐Spi9 ranked by z‐score. **C**. Heatmap of normalized reads of top DEGs with main biological processes involved in control and LV‐US2‐Spi9‐transduced leaflets analysed using DESeq2 differential expression analysis. Data are shown as the mean ± s.e.m. * *p* < 0.05; ** *p* < 0.01; compared to control using one‐way ANOVA and Dunnett multiple comparison.

## 4 Conclusions

Living heart valves hold immense potential in achieving the highest durability and best outcome in young patients requiring a heart valve replacement, as illustrated by the Ross procedure and partial heart transplantations. However, living donor heart valves face a risk of immune rejection. Cardiovascular tissue engineering has made numerous promising steps in order to create a non‐immunogenic living valve with self‐repair and remodelling capacity. Despite much effort in the scientific field, in situ tissue engineering has not yet succeeded in creating a valve with these characteristics due to the difficulty of harnessing the delicate balance of the immune system in regulating either neo‐tissue formation or valve degeneration (retraction of the leaflets, calcification of the valve) [109]. The issue of immune responses triggered by the implantation of grafts intended to replace diseased tissues extends beyond the field of heart valves. The intermingled network of all the components of the immune system makes it extremely complicated to identify which elements or cells drive which responses. In transplantation, nonself HLA molecules are considered the main drivers of immune rejection [110]. In previous work, we showed viral immune evasion strategies could be harnessed to reduce the expression of donor HLA molecules on the cell surface [42].

In the present study, we demonstrate that delivery of viral immune evasion genes US2 and Serpin b9 significantly reduces PBMC‐induced cell death in both VICs and VECs. Notably, the extent of immune‐mediated cell death in untransduced controls correlated with the degree of HLA mismatch between donor and recipient. A similar pattern was reflected in our fresh whole‐tissue cocultures, where one donor combination showed a markedly higher Granzyme B response than the others. Fresh human heart valve tissue is a scarce and valuable resource, and we prioritized using all available donor material for transduction and immune cell coculture assays rather than diverting tissue for HLA typing. Consequently, donor–PBMC HLA mismatch could not be confirmed as the cause of the higher Granzyme B levels in this donor although that would be in line with our findings in both isolated valvular cell types. Despite the higher baseline in this donor, US2/Spi9 transduction still reduced Granzyme B in the same direction as all other combinations, supporting the robustness of the effect across a heterogeneous donor cohort. Of note, our epitope analyses are solely focused on the indirect allorecognition pathway (PIRCHE‐T2) and direct B‐cell recognition of intact donor HLA (PIRCHE‐B). Direct recognition of donor HLA by CD8^+^ T cells could not be assessed, as no validated in silico algorithm currently exists for this pathway, given its dependence on T‐cell receptor cross‐reactivity to intact, unprocessed donor HLA‐peptide complexes rather than defined linear epitopes. Nonetheless, the PIRCHE‐T2 score used here has been shown to associate with both T‐cell‐mediated rejection and de novo donor‐specific antibody development in kidney transplantation, suggesting it captures risk relevant to both cellular and humoral arms of alloimmunity, despite modelling only the indirect pathway [64, 111]. In clinical practice, HLA matching is rarely feasible when selecting donor heart valves, as anatomical fit, particularly valve diameter, and valve availability are the primary considerations. Additionally, low cellular viability of cryopreserved allografts precludes any meaningful contribution of donor cells to tissue repair or growth while cellular debris can cause immune activation. Consequently, HLA matching is not standard practice. Precisely, due to this, decellularization of cryopreserved allografts, and thereby removing potentially immune triggering cellular debris, has gained much interest and has shown successful results in clinical trials in adults [112, 113]. However, the absence of living cells capable of growth and remodelling limits their durability in pediatric patients.

Given the constraints on HLA matching, developing strategies to improve immune compatibility remains a critical goal. Such approaches could be particularly valuable in partial heart transplantation or in emerging techniques designed to implant homovital (viable) valves. Alongside our decreased immune‐induced valve cell loss in LV‐US2‐Spi9 transduced heart valve cells, we also observed immune responses to heart valve tissue decreased following LV‐US2‐Spi9 transduction. Bulk RNA sequencing further revealed that transduced cells did not exhibit activation of cell damage or death pathways. The pathways that were altered after LV‐US2‐Spi9 delivery were associated with the process of antigen presentation and immune activation. Unexpectedly, we observed an upregulation of mRNA transcripts associated with antigen presentation pathways. Although this did not translate into higher HLA surface expression, it may represent a compensatory cellular response to the downregulation of HLA proteins by US2. While we have previously shown stable US2 transgene expression 8 weeks after transduction [15], this phenomenon raises important questions about how cells adapt to immune evasion strategies over time and how these adaptations affect long‐term immunogenicity. Interestingly, our RNA sequencing dataset showed transduction with US2 and Serpin b9 stimulates or inhibits genes previously described in literature as aiding immune evasion in cancer cells, viruses or the feto‐maternal interface. It is important to note that with 7‐day coculture, our immune assay primarily mimics lymphocyte effector responses as myeloid cell survival might be unlikely at this timepoint. Monocytes (in vitro and circulating in vivo) normally live 24–48 h and, in the absence of a survival signal, undergo spontaneous apoptosis [114, 115]. Our immune‐shielding strategy was specifically developed to target lymphocyte‐mediated, not monocyte‐mediated, immune responses, to address previous findings that mainly lymphocytes drive heart allograft rejection [45, 46, 116, 117, 118]. CD4^+^ T cells have been described as both necessary and sufficient to trigger acute cardiac allograft rejection, while the inability of graft cells to provide HLA class I‐mediated inhibitory signals to recipient NK cells triggers their activation and promotes endothelial damage [119, 120, 121, 122]. Monocytes, while contributing to the inflammatory milieu, are not direct cytotoxic effectors of valve cell killing [123, 124, 125] and are not targeted by our shielding constructs. While our in vitro immune assays mimics T‐cell‐mediated immune responses, our sequencing dataset shows US2 and Serpin b9 delivery also alter gene expression associated with B‐cell responses [76, 82]. Future studies will be necessary to determine the functional consequences of these transcriptional changes and assess whether the observed compensatory responses impact the longevity and integration of engineered heart valve grafts in vivo. The ultimate goal is to support the development of viable heart implants for truncal valve replacements in neonates and to provide valuable insight for the broader field of transplantation medicine.

## Author Contributions

**Abraham van Wijk**: conceptualization, funding acquisition, writing – review and editing, and supervision. **Leanne Van den Berg**: investigation. **Valentina Osorio Zuluaga**: investigation. **Arnaud Zaldumbide**: methodology and resources. **Rob C. Hoeben**: conceptualization, methodology, resources, and supervision. **Eric Spierings**: methodology, resources, and writing – review and editing. **Marijn C. Peters**: conceptualization, investigation, funding acquisition, writing – original draft, methodology, visualization, writing – review and editing, project administration, and formal analysis.

## Funding

Financial support from the Gravitation Program “Materials Driven Regeneration” (to MCP and CB) and ENW‐XS grant (to MCP) funded by the Dutch Research Council (NWO) (024.003.013; OCENW.XS23.2.077) and Hartekind Foundation as part of the OUTREACH Netherlands Heart Institute (NLHI) grant (to MCP, AvW) is gratefully acknowledged.

## Ethics Statement

Aortic and pulmonary valves were obtained from patients undergoing surgical valve replacement at Wilhelmina Children's Hospital at Utrecht University Medical Center (Utrecht, the Netherlands). Tissue was obtained in accordance with the Declaration of Helsinki protocols and approved by the medical ethical assessment committee of Wilhelmina Children's Hospital at Utrecht University Medical Center (Utrecht, the Netherlands, TCBio number: 22–828).

## Consent

For pediatric valves, informed consent was obtained from parents or legal guardians. Additionally, male and female aortic and pulmonary valves were obtained from donor hearts processed by the Heart Valve Department of multi‐tissue bank ETB‐BISLIFE (Beverwijk, the Netherlands). Donor mediation took place via The Dutch Transplant Foundation (Leiden, the Netherlands), and permission for transplantation‐related research was given by each donor.

## Conflicts of Interest

The University Medical Center Utrecht has filed patents/patent applications for the prediction of an alloimmune response against mismatched HLA. E.S. is listed as inventor on this patent. The other authors declare no conflicts of interest.

## Supporting information




**Supporting File 1**: adhm71737‐sup‐0001‐SuppMat.pdf.

**Supporting File 2**: adhm71737‐sup‐0002‐SuppMat.docx.

## Data Availability

The data that support the findings of this study are available from the corresponding author upon reasonable request. The data are not publicly available due to privacy or ethical restrictions.
