# Supplementary material for: Comprehensive In Vitro Profiling Demonstrates Successful Immune Evasion of Bioengineered Immune‐Shielded Heart Valves
Source: Adv Healthc Mater. 2026 Sep 21;15(39):e71737. doi: 10.1002/adhm.71737 (PMC13691386; doi:10.1002/adhm.71737)
Supplement: Supplementary file 1 [file ADHM-15-0-s002.pdf]

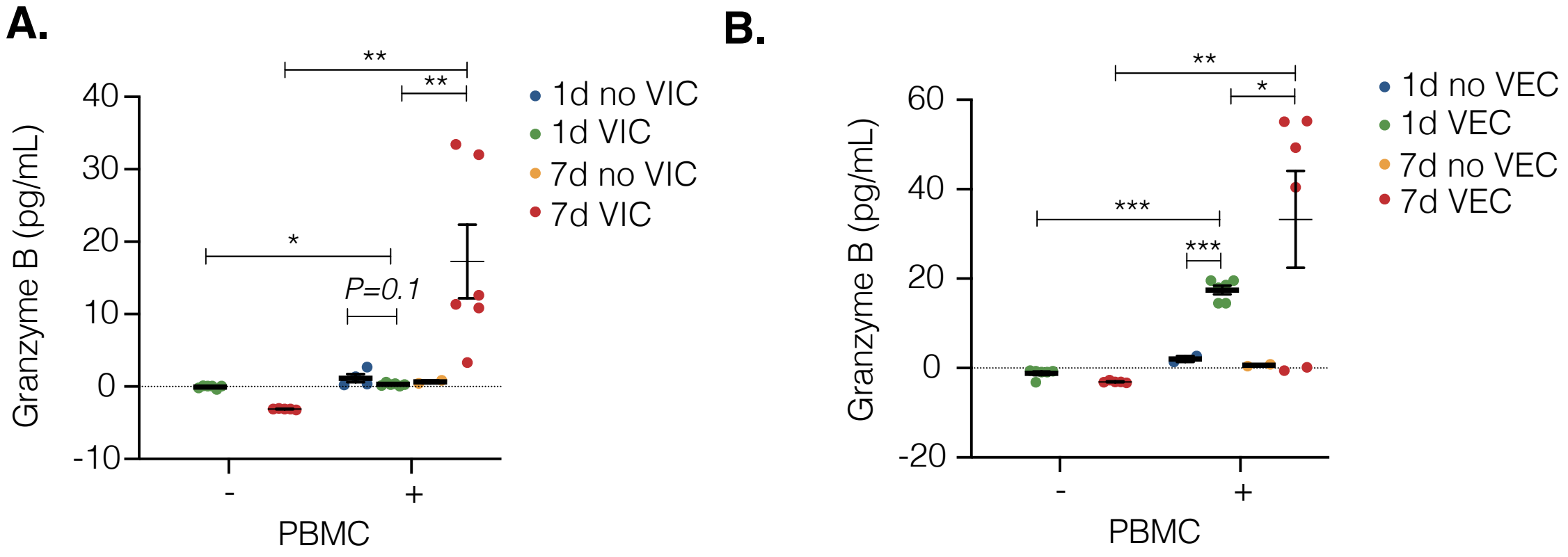

Supplemental figure 1: Granzyme B release in PBMC-valve cell 1-day and 7-day co-culture.
A,B. Granzyme B levels in conditioned media of 1-day and 7 day VIC (A) or VEC (B) monoculture or PBMC co-cultures. Data are shown as the mean ± SEM. * P<0,05, ** P<0.01, ***P<0.001 compared to control using one-way ANOVA and Dunnett's multiple comparisons test.

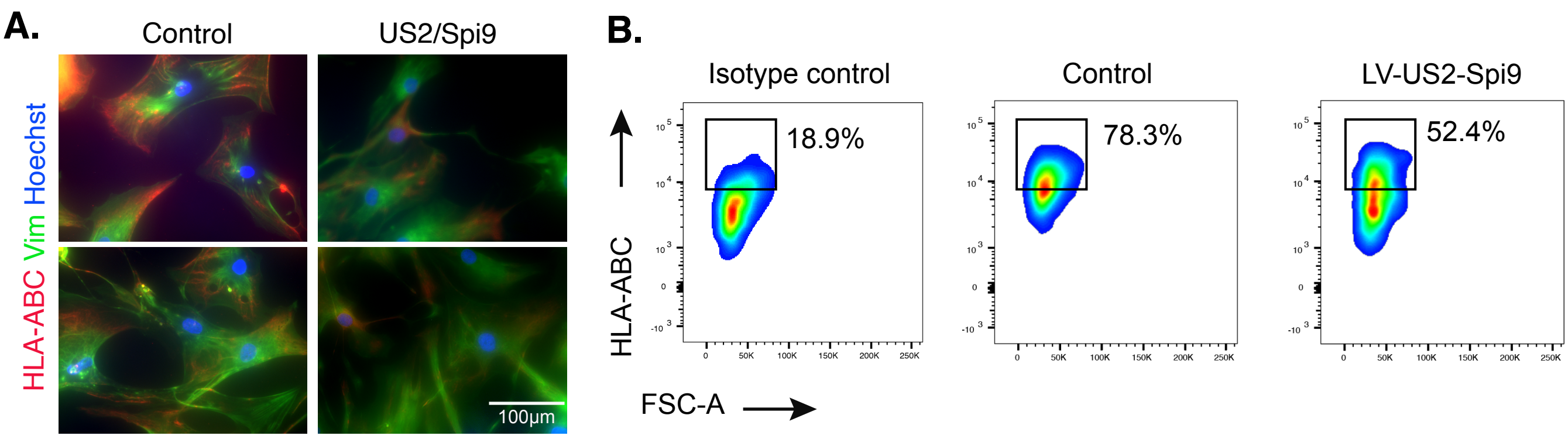

Supplemental figure 2: Cell surface HLA-ABC expression in control and LV-US2-Spi9 transduced VICs .
A. Validation of transduction efficiency immunofluorescence imaging of polyclonal antibody against HLA-A, HLA-B, HLA-C showing decreased HLA levels following transduction. **B**. Flow cytometric plots of HLA-ABC staining in control and LV-US2-Spi9 transduced human VICs.

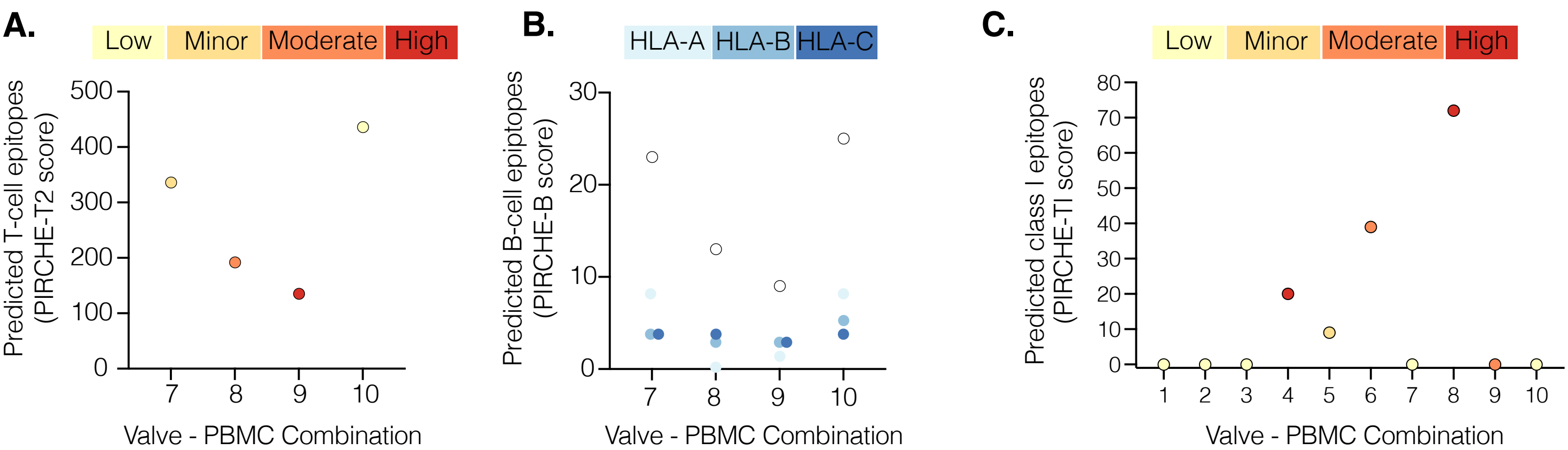

Supplemental figure 3: Predicted T and B cell epitopes of valve cell-immune cell combinations.
A.PIRCHE-T2 scores of predicted T-cell epitopes in four VEC-PBMC combinations. **B**. Total PIRCHE-B score and PIRCHE-B score for HLA-A, HLA-B, and HLA-C are shown for four VEC-PBMC combinations. **C**. PIRCHE-TI scores predicting epitopes derived from mismatched donor HLA molecules that could be presented on the recipient's HLA class I molecules to CD8+ T cells.

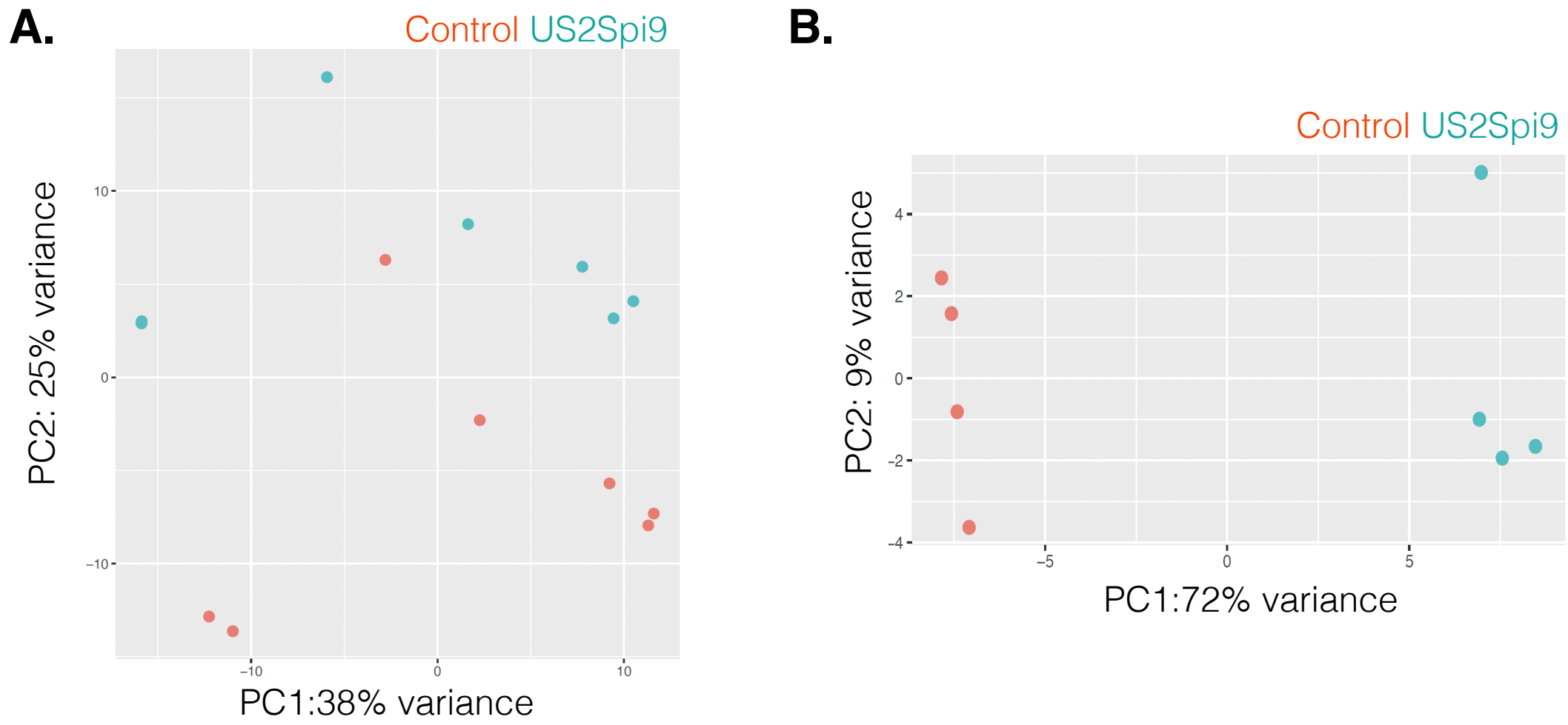

Supplemental figure 4: Principal Component Analysis of Control and US2/Spi9 treated VECs and VICs
A, B. Principal Component Analysis (PCA) plots of US2/Spi9 transduced VICs (A) and VECs (B) and control cells showing the separation between the samples based on the top differentially expressed genes using DESEQ2.

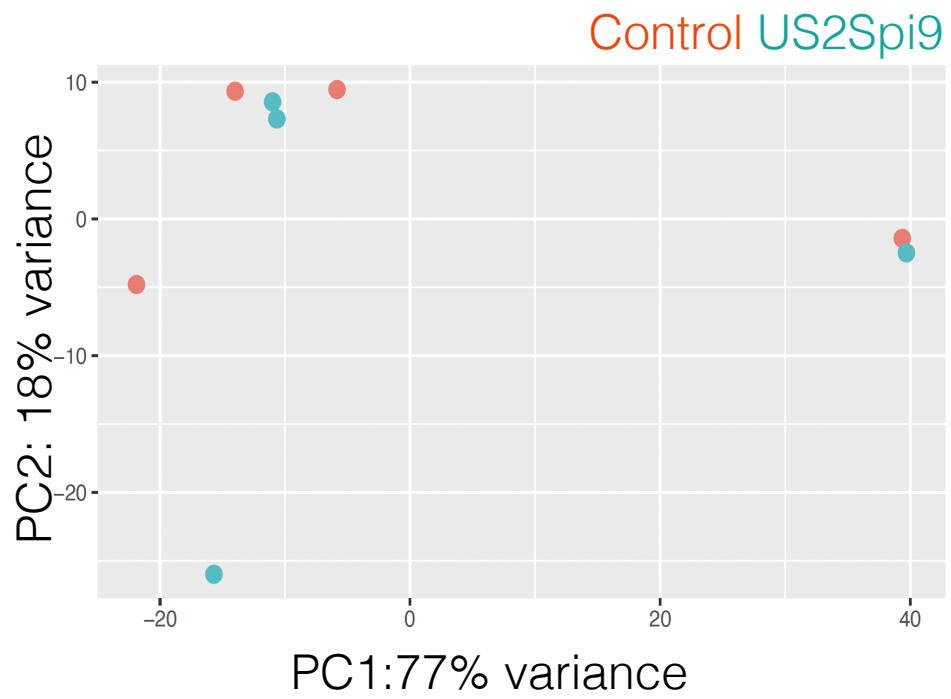

Supplemental figure 5: Principal Component Analysis of Control and US2/Spi9 treated heart valves
Principal Component Analysis (PCA) plots of US2/Spi9 transduced heart valves and control valves showing the separation between the samples based on the top differentially expressed genes using DESEQ2.
