# Supplementary material for: Comprehensive In Vitro Profiling Demonstrates Successful Immune Evasion of Bioengineered Immune‐Shielded Heart Valves
Source: Adv Healthc Mater. 2026 Sep 21;15(39):e71737. doi: 10.1002/adhm.71737 (PMC13691386; doi:10.1002/adhm.71737)
Supplement: Supplementary file 2 [file ADHM-15-0-s001.docx]

Supplementary information

**Supplementary table 1: Top enriched Gene Ontology (GO) terms among differentially expressed genes.** Gene Ontology enrichment analysis was performed on the set of differentially expressed genes (DEGs; |log2FC| > 1, adjusted p < 0.05). GO ID and Term indicate the identifier and description of each enriched biological process (BP) category. Z-score was calculated as (up − down)/√count, where "up" and "down" represent the number of upregulated and downregulated genes within the term, respectively, providing an indication of the overall direction of regulation. Adjusted p-values were calculated using the Benjamini-Hochberg method to correct for multiple testing. Representative genes contributing to each term are listed under Top Genes. Only terms with adjusted p < 0.05 are shown.

| **GO ID** | **Term** | **Cell type** | **Z-score** | **Top genes** |
| --- | --- | --- | --- | --- |
| GO:0002720 | Cytokine Production in Immune Response | VIC | -1,73 | HLA-F, MAP3K7, CD55 |
| GO:0048525 | Negative Regulation of Viral Process | VIC | 6,868 | IRF7, OAS1, IL1B, ICAM1, RSAD2, IL32, IFITM1, RSAD2, MX1, ISG15 |
| GO:0030968 | Endoplasmatic Reticulum Unfolded Protein Response | VIC | 3,606 | CREBZF,PTPN1, HSPA5, SERP1, DERL |
| GO:0045824 | Negative Regulation of Innate Immune Response | VIC | 2,450 | DDX39A, OAS1, YTHDF3, OAS3, SG15, USP18 |
| GO:0002286 | T-cell Activation | VIC | 2,450 | PLXNA1, ICAM1 |
| GO:0048525 | Negative Regulation of Viral Process | VEC | 8,124 | GRB2, LGALS9, IFITM1, SP100, IFITM2, OASL, |
| GO:0045824 | Negative Regulation of Innate Immune Response | VEC | 4,025 | GRN, YTHDF2, PARP1, YTHDF3, ISG15 |
| GO:0002709 | Regulation of T-cell Mediated Immunity | VEC | 3,051 | HLA-A, HLA-B, HLA-C, B2M, CD46 |
| GO:2001242 | Regulation of Intrinsic Apoptotic Signalling Pathway | VEC | 2,324 | E2F1, TNFRSF1B |
| GO:0002716 | Negative Regulation of NK Cell Mediated Immunity | VEC | 1,732 | GRB2, LGALS9, HLA-F, AK1, MIA3, CD200 |
| GO:0042270 | Protection from Natural Killer Cell Mediated Cytotoxicity | Valve | 0,707 | HLA-E, HLA-DOA, TIMP1 |
| GO:0043161 | Proteasome-Mediated Ubiquitin-Dependent Protein Catabolic Process | Valve | 1,732 | PSMB8, PSMB9 |
| GO:0002684 | Positive Regulation of Immune System Process | Valve | -0,577 | TNFSF18, FCAR |
| GO:0002446 | Neutrophil Mediated Immunity | Valve | -1,732 | ANXA3, FCAR |
| GO:0002092 | Positive Regulation of Receptor Internalization | Valve | -1,732 | GREM1, APLNR |
